# Prelacteal feeding is not associated with infant size at 3 months in rural Bangladesh: a prospective cohort study

**DOI:** 10.1186/s13006-024-00621-4

**Published:** 2024-02-27

**Authors:** Hannah Tong, Andrew Thorne-Lyman, Amanda C. Palmer, Saijuddin Shaikh, Hasmot Ali, Ya Gao, Monica M. Pasqualino, Lee Wu, Kelsey Alland, Kerry Schulze, Keith P. West, Jr., Md Iqbal Hossain, Alain B. Labrique

**Affiliations:** 1 Department of International Health, Johns Hopkins Bloomberg School of Public Health, Baltimore, MD USA; 2 JiVitA Project, Johns Hopkins University, Bangladesh (JHU,B) Keranipara, Rangpur, Bangladesh; 3 International Centre for Diarrhoeal Disease Research, Dhaka, Bangladesh

**Keywords:** Breastfeeding, Neonatal health, Infant development, Prelacteal, Early newborn food, South Asia

## Abstract

**Background:**

Early and exclusive breastfeeding may reduce neonatal and post-neonatal mortality in low-resource settings. However, prelacteal feeding (PLF), the practice of giving food or liquid before breastfeeding is established, is still a barrier to optimal breastfeeding practices in many South Asian countries. We used a prospective cohort study to assess the association between feeding non-breastmilk food or liquid in the first three days of life and infant size at 3–5 months of age.

**Methods:**

The analysis used data from 3,332 mother-infant pairs enrolled in a randomized controlled trial in northwestern rural Bangladesh conducted from 2018 to 2019. Trained interviewers visited women in their households during pregnancy to collect sociodemographic data. Project staff were notified of a birth by telephone and interviewers visited the home within approximately three days and three months post-partum. At each visit, interviewers collected data on breastfeeding practices and anthropometric measures. Infant length and weight measurements were used to produce length-for-age (LAZ), weight-for-age (WAZ), and weight-for-length (WLZ) Z-scores. We used multiple linear regression to assess the association between anthropometric indices and PLF practices, controlling for household wealth, maternal age, weight, education, occupation, and infant age, sex, and neonatal sizes.

**Results:**

The prevalence of PLF was 23%. Compared to infants who did not receive PLF, infants who received PLF may have a higher LAZ (Mean difference (MD) = 0.02 [95% CI: -0.04, 0.08]) score, a lower WLZ (MD=-0.06 [95% CI: -0.15, 0.03]) score, and a lower WAZ (MD=-0.02 [95% CI: -0.08, 0.05]) score at 3–5 months of age, but none of the differences were statistically significant. In the adjusted model, female sex, larger size during the neonatal period, higher maternal education, and wealthier households were associated with larger infant size.

**Conclusion:**

PLF was a common practice in this setting. Although no association between PLF and infant growth was identified, we cannot ignore the potential harm posed by PLF. Future studies could assess infant size at an earlier time point, such as 1-month postpartum, or use longitudinal data to assess more subtle differences in growth trajectories with PLF.

**Trial registration:**

ClinicalTrials.gov: NCT03683667 and NCT02909179.

**Supplementary Information:**

The online version contains supplementary material available at 10.1186/s13006-024-00621-4.

## Background

Age-appropriate breastfeeding practices provide many health benefits for infants and young children in low- and middle- income countries (LMICs), including reducing risk of infection [1–3], malnutrition [4, 5], and under-five mortality [6–8]. The World Health Organization (WHO) recommends exclusive breastfeeding for infants under six months of age and early initiation of breastfeeding within one hour of birth [9]. Breastmilk not only contains high quality macro- and micro-nutrients necessary for growth but also many distinct bioactive molecules that protect against infection and inflammation and contribute to immune maturation, organ development and healthy microbial colonization [10].

Despite the proven benefits and active advocacy efforts, adherence to age-appropriate breastfeeding remains low in many LMICs. Only half of newborn babies are put to the breast within the first hour of life and about one in three neonates in LMICs receive prelacteal feed substances during the first three days after birth [11]. In Bangladesh and other South Asia countries, prelacteal feeding (PLF), defined as “giving a newborn baby any food or liquids before breastfeeding is established” [12], is a major barrier to age-appropriate breastfeeding. Based on the 2019 Bangladesh Multiple Indicator Cluster Survey (MICS), it was estimated that only about 47% infants were breastfed within one hour of birth and only 63% infants under 6 months of age were exclusively breastfed [13]. There are two main reasons why PLF is common in South Asia. One is traditional beliefs, including a perception that feeding something sweet to the newborns is associated with strength or better luck in the future [14–16]. The other reason is limited knowledge about breastfeeding, ranging from negative perceptions of colostrum [17], not knowing how to position the baby while breastfeeding [18], to perceiving milk production as insufficient [16]. The most recent national estimate on prevalence of exposure to PLF within first three days of life in Bangladesh was 24% in 2019 [13].

Previous studies have shown that PLF delayed breastfeeding initiation [14, 19] and disrupted exclusive breastfeeding [20–22]. The early life exposure to non-breastmilk supplements might also introduce environmental contaminants [23, 24] and interfere with breastmilk’s protection against potential insults [25]. The disruption of exclusive breastfeeding and introduction of contaminants will likely put infants at higher risk of infections like diarrhea incidence, which impairs linear growth.

In many surveys, a one-day recall is used to classify breastfeeding practices at a population level. The data collected on exclusive breastfeeding lack sensitivity, in which children who consumed non-breastmilk liquid or food prior to the survey could be classified as exclusive breast feeding. This overestimation of EBF rates by 24-hour recall measure has been observed previously [26, 27].

Given the definition of PLF, the one-day recall survey failed to capture exposure to PLF, and the potential harm of PLF thus remains underappreciated. In addition, the cross-sectional nature of current studies lacks the ability to capture feeding practices as they occur right after birth. To our knowledge no studies have examined the impact of PLF on infant size using a prospective study design. The objective of this analysis was to explore the association between feeding non-breastmilk food or liquid in the first three days of life and infant size at 3 to 5 months using data from a prospective cohort of infants.

## Methods

### Study setting and procedure

Data for this analysis were collected at the JiVitA Research Site located in Gaibandha District in Rangpur Division in northwestern Bangladesh. The site has hosted several studies of maternal and child health [28–32].

A pregnancy surveillance system was launched in the site in June 2016 as part of the protocol for an mHealth Intervention trial (mCARE-II; NCT02909179). We surveyed households in the study area to identify all married women of reproductive age and obtain their consent for pregnancy surveillance. Field staff then visited households every two months to ask about their last menstrual period. Women who became pregnant were recruited into the mCARE-II trial, which documented sociodemographic, birth outcomes, and the health and nutrition of women and infants to 1 month postpartum. Infants born to women enrolled in mCARE-II who survived and whose families were still living in the study area at three months of age during the period from September 2018 through July 2019 were considered eligible for enrollment in a cluster-randomized controlled protein supplementation trial (NCT03683667) designed to address linear growth faltering in 6–12-month-old infants, hereafter referred to as JiVitA-6. Thus, enrollment for the JiVitA-6 trial was nested within the mCARE-II trial framework.

For women and infants enrolled in the JiVitA-6 trial, field staff visited the household during pregnancy, within three days post-partum (hereafter referred to as the “birth visit”), and at three months to conduct structured interviews.

The final analyses included singleton live births. To reduce the risk of recall bias in responses, we limited the analysis to interviews conducted within 14 days of the scheduled birth visit and 60 days of the scheduled 3-mo visit [see Additional file 1 (Supplementary Fig. 1)].

### Exposure

The information on PLF was collected during the birth visit, where information was collected from birth up to 3 days of life. Mother were asked: “Was the baby fed other mother’s breast milk or anything than own mother’s breastmilk in the [first 30 minutes, second 30 minutes, 2nd hour, 3rd hour, 4th hour, 5th hour, 6th hour, 7th-12th hours, 13th-24th hours, remaining hours until upcoming 6 am after completion of 24-hour, entire day 3 and night]?”

For each time interval, if the mother responded yes, data on feeding from other mother’s milk or specific foods from a list of common non-breast milk feeds (honey, water, animal milk, formula, sugar/sugar candy water, any types of drops, power/condensed milk, and others) were also collected.

### Outcomes

Infant weight and length were measured by trained field staff according to standardized protocols at the birth visit and at three months of age. Undressed infants were weighed to the nearest 0.01 kg using a Tanita BD-585 Pediatric Scale. Scales were calibrated daily with 2.5 kg, 5 kg, and 10 kg weights. Infant recumbent length was measured to the nearest 0.1 cm three times on a measuring board with a sliding foot piece.

### Statistical analysis

Exposure to PLF was dichotomized into responses “yes” and “no”, where “yes” was defined as reporting one or more items from the list of common non-breast milk feeds within 3 days of life at the birth visit.

The medium value was used as the final measure of infant length. Infant length and weight measurements were used to produce length-for-age (LAZ), weight-for-length (WLZ), and weight-for-age (WAZ) Z-scores using the sex-specific WHO standards [33]. Stunting, wasting, and underweight were defined as a LAZ, WLZ, and WAZ <-2, respectively. In the final analysis, we also excluded any anthropometric measures that were outside normal bounds, based on a Z-score beyond ± 6 [see Additional file 1 (Supplementary Fig. 1)] (Supplement Fig. 1).

The potential confounding variables were chosen based on previous observational studies that examined factors associated with infant growth [34–38]. The selected covariates were neonate size, infant sex, infant age, maternal age, maternal BMI, maternal education, maternal occupation, and household wealth. LAZ and WAZ collected at birth visits were used as indicators for neonatal size. Maternal occupation was coded as “yes” or “no” to represent maternal employment status. Maternal BMI was calculated using maternal weight and height collected at the 3-month postpartum visit. A composite Living Standards Index (LSI) based on housing material and durable asset ownership was calculated as previously described [39]. LSI, categorized into five quintiles (1–5, corresponding to the poorest to the wealthiest groups), was used as an indicator for household wealth. Categorical variables were presented as percent (n) and continuous variables presented as mean ± SD.

We used simple linear regression to assess the association between PLF and anthropometric indices (LAZ, WLZ and WAZ). We also used multiple linear regression to assess the association between PLF and anthropometric indices (LAZ, WLZ and WAZ), controlling for other covariates. All data management and statistical analysis were conducted in STATA version 15.1. Infants with missing PLF responses or anthropometric measures were excluded from the analysis [see Additional file 1 (Supplementary Fig. 1)] (Supplement Fig. 1). None of the covariates included in the multivariate models had variance inflation factors higher than 2.5, suggesting that there was no observed multicollinearity among covariates in the final models.

## Results

A total of 3,332 mother and infant pairs were included in the final analysis [see Additional file 1 (Supplementary Fig. 1)] (Supplement Fig. 1). Characteristics of mothers and infants broken out by PLF status and combined are shown in Table 1. The majority of the mothers were between 20 and 29 years old (58.3%), attended school from class one to nine (73.0%), and had no occupation (70.1%). The average neonatal weight and length during birth visit were 2.8 ± 0.4 kg and 47.9 ± 2.2 cm, respectively. 25% of the infants received a birth visit on the first day of life and 75% of infants received a birth visit on the eighth day of life. The prevalence of stunting, wasting, and underweight at the three-month visit were 20.3%, 3.9%, and 16.2%, respectively. 25% of infants received a three-month visit at 3.1 months of life and 75% of infants received a three-month visit at 3.83 months of life (Table 1).

**Table 1:** Prelacteal feeding and demographic characteristics of women and infants in rural Bangladesh

	No Prelacteal Feeding (*n* = 2,583)	Prelacteal Feeding (*n* = 749)	Total (*n* = 3,332)
	*n* (%) or Mean ± SD	*n* (%) or Mean ± SD	*n* (%) or Mean ± SD
*Household*			
Wealth quintile			
1	515 (20.3)	146 (19.8)	661 (20.2)
2	509 (20.0)	144 (19.5)	653 (19.9)
3	521 (20.5)	136 (18.4)	657 (20.0)
4	507 (20.0)	149 (20.2)	656 (20.0)
5	490 (19.3)	164 (22.2)	654 (19.9)
*Maternal*			
Age (years)			
≤19	619 (24.5)	190 (25.8)	809 (24.8)
20–29	1486 (58.7)	419 (56.8)	1905 (58.3)
>29	425 (16.8)	129 (17.4)	554 (17.0)
BMI (kg/m^2^)	21.57 ± 3.18†	21.96 ± 3.34	21.66 ± 3.22
Education			
No schooling	300 (11.8)	90 (12.2)	390 (11.9)
Class 1 to 9	1868 (73.6)	521 (70.8)	2389 (73.0)
10 years and above	370 (14.6)	125 (17.0)	495 (15.1)
Occupation^a^			
Yes	764 (30.1% (764)	216 (29.2)	980 (29.9)
No	1778 (69.9)	523 (70.8)	2301 (70.1)
*Infant*			
Female	1269 (49.1)	331 (44.2)	1600 (48.0)
Neonatal measures			
Weight (kg)	2.78 ± 0.43	2.78 ± 0.45	2.78 ± 0.43
Length (cm)	47.90 ± 2.20	47.95 ± 2.27	47.91 ± 2.22
WAZ	-1.32 ± 0.96	-1.34 ± 1.06	-1.32 ± 0.99
LAZ	-1.26 ± 1.08	-1.27 ± 1.14	-1.26 ± 1.10
3-month measures			
Stunting	535 (20.7)	140 (18.7)	675 (20.3)
Wasting	96 (3.7)	34 (4.5)	130 (3.9)
Underweight	412 (16.0)	127 (17.0)	539 (16.2)
Birth visit^b^ (days)			
25th percentile	1.00	1.00	1.00
75th percentile	7.00	8.00	8.00
3-month visit^b^ (months)			
25th percentile	3.10	3.10	3.10
75th percentile	3.83	3.83	3.83

LAZ: Length-for-age Z-score; WLZ: Weight-for-length Z-score; WAZ: Weight-for-age Z-score; BMI: body mass index

Birth Visit is designed to be completed within first 3 days of life

^a^ In the interview, occupation is defined as working on own farm/as sharecropper; or Day, unskilled laborer (agriculture & migrant etc.); or Maid servant/Fisherman; or Contracted laborer (long term domestic, agricultural); or Own business; or Private service (salaried, skilled factory and office workers etc.); or Government service; or Other, specified

^b^ Present the 25th and 75th percentiles because the distributions are skewed

PLF were provided to 749 (22.5%) infants (Table 1). The most common PLFs were animal milk (5.1%), followed by honey (5.0%), sugar water (5.0%), any types of drops (3.6%), and formula (2.9%) (Table 2). Among the infants who received PLF, 649 (86.6%) infants received only one type of PLF, and 100 (13.4%) infants received two or more types of PLF.

**Table 2:** Frequency of prelacteal feeding by types among mother and infant pairs in rural Bangladesh (*N* = 3,332)

Types of prelacteal feeding	Number of Infants receiving *n* (%)
Honey	168 (5.0)
Water	13 (0.4)
Animal milk	171 (5.1)
Formula	98 (2.9)
Sugar/Sugar candy water	165 (5.0)
Drops, unspecified	121 (3.6)
Powder/Condensed milk	37 (1.1)
Other, unspecified	83 (2.5)

Note: The frequencies shown for an individual prelacteal feed are nonexclusive, meaning that it was possible for one woman to feed two or more types of foods

In both unadjusted and adjusted analyses (Table 3), there was no association between PLF and infant LAZ, WAZ, and WLZ at 3 months of age, and point estimates for the association of PLF with anthropometry at 3 months of age were virtually unchanged between the unadjusted and adjusted models, suggesting that none of the covariates were confounding an association between PLF and size at 3 months.

**Table 3:** Regression model to assess the relationship between prelacteal feeding and LAZ, WLZ, WAZ at 3–5 months of age

	LAZ (*n* = 3,176)		WLZ (*n* = 3,176)		WAZ (*n* = 3,176)	
	UnadjustedCoefficient (95% CI)	Adjusted Coefficient^a^ (95% CI)	Unadjusted Coefficient (95% CI)	Adjusted Coefficient^b^ (95% CI)	Unadjusted Coefficient (95% CI)	Adjusted Coefficient^b^ (95% CI)
*Prelacteal foods*						
No	Reference	Reference	Reference	Reference	Reference	Reference
Yes	0.02 (-0.07, 0.11)	0.02 (-0.04, 0.08)	-0.05 (-0.14, 0.04)	-0.06 (-0.15, 0.03)	-0.01 (-0.10, 0.07)	-0.02 (-0.08, 0.05)
*Household*						
Wealth quintile						
1	Reference	Reference	Reference	Reference	Reference	Reference
2	0.17 (0.06, 0.29)††	0.07 (-0.01, 0.15)	-0.02 (-0.14, 0.10)	-0.04 (-0.16, 0.08)	0.12 (0.01, 0.23) †	0.03 (-0.06, 0.12)
3	0.23 (0.11, 0.34)††	0.12 (0.04, 0.21)††	0.05 (-0.07, 0.17)	0.01 (-0.12, 0.13)	0.22 (0.11, 0.33)††	0.09 (0.00, 0.18)†
4	0.17 (0.05, 0.28)††	0.09 (0.01, 0.18)†	0.04 (-0.08, 0.16)	0.00 (-0.12, 0.13)	0.17 (0.07, 0.28)††	0.08 (-0.01, 0.17)
5	0.36 (0.25, 0.48)††	0.20 (0.12, 0.30)††	0.01 (-0.11, 0.13)	-0.04 (-0.17, 0.10)	0.30 (0.19, 0.41)††	0.11 (0.01, 0.21)†
*Maternal*						
Age (years)						
≤19	Reference	Reference	Reference	Reference	Reference	Reference
20–29	0.15 (0.06, 0.24)††	0.04 (-0.02, 0.11)	0.03 (-0.06, 0.12)	-0.03 (-0.13, 0.07)	0.14 (0.06, 0.22)††	0.00 (-0.07, 0.07)
>29	0.03 (-0.08, 0.15)	0.05 (-0.03, 0.14)	-0.05 (-0.17, 0.07)	-0.11 (-0.24, 0.02)	0.00 (-0.11, 0.10)	-0.04 (-0.13, 0.06)
BMI	0.04 (0.03, 0.05)††	0.00 (-0.01, 0.01)	0.03 (0.01, 0.04)††	0.02 (0.01, 0.04)††	0.05 (0.04, 0.06) ††	0.01 (0.00, 0.02)
Education						
No schooling	Reference	Reference	Reference	Reference	Reference	Reference
Class 1 to 9	0.21 (0.09, 0.33)††	0.03 (-0.06, 0.11)	0.08 (-0.04, 0.20)	0.04 (-0.08, 0.17)	0.24 (0.13, 0.34) ††	0.07 (-0.02, 0.16)
10 years and above	0.44 (0.30, 0.58)††	0.07 (-0.04,0.18)	0.05 (-0.10, 0.19)	0.00 (-0.16, 0.17)	0.40 (0.27, 0.53)††	0.15 (0.03, 0.27)†
Occupation^c^						
Yes	Reference	Reference	Reference	Reference	Reference	Reference
No	0.13 (0.05, 0.21)††	0.01 (-0.05, 0.06)	0.00 (-0.09, 0.08)	-0.01 (-0.09, 0.08)	0.10 (0.03, 0.18) ††	0.01 (-0.05, 0.08)
*Infant*						
Age (months)	0.03 (-0.04, 0.10)	-0.02 (-0.07, 0.02)	-0.10 (-0.17,-0.03)††	-0.11 (-0.18, -0.04)††	0.03 (-0.03. 0.10)	-0.01 (-0.06, 0.05)
Sex						
Male	Reference	Reference	Reference	Reference	Reference	Reference
Female	0.19 (0.12, 0.26)††	0.11 (0.06, 0.16)††	-0.06 (-0.13, 0.02)	-0.07 (-0.14, 0.01)	0.06 (0.00, 0.13)	0.01(-0.04, 0.07)
Neonatal measures						
WAZ			0.08 (0.04, 0.12)††	0.07 (0.03, 0.11)††	0.08 (0.04, 0.12)††	0.60 (0.57, 0.63)††
LAZ	0.71 (0.70, 0.74)††	0.71 (0.69, 0.74)††				

LAZ: Length-for-age Z-score; WLZ: Weight-for-length Z-score; WAZ: Weight-for-age Z-score; BMI: body mass index

^a^ Multiple linear regression model adjusted for infant age, infant sex, neonatal LAZ, maternal age, maternal education, maternal occupation, maternal BMI, living standard index

^b^ Multiple linear regression model adjusted for infant age, infant sex, neonatal WAZ, maternal age, maternal education, maternal occupation, maternal BMI, living standard index

^c^ In the interview, occupation is defined as working on own farm/as sharecropper; or Day, unskilled laborer (agriculture & migrant etc.); or Maid servant/Fisherman; or Contracted laborer (long term domestic, agricultural); or Own business; or Private service (salaried, skilled factory and office workers etc.); or Government service; or Other, specified

†Statistically significant at *P* < 0.05

††Statistically significant at *P* < 0.01

Other factors were found to be associated with infant anthropometry at 3 months of age in the adjusted model. Higher neonatal LAZ (β = 0.71; 95% CI: 0.69, 0.74), female infant (β = 0.11; 95% CI: 0.06, 0.16), and higher household wealth quintile were positively associated with LAZ at 3–5 months of life. Higher neonatal WAZ (β = 0.60; 95% CI: 0.57, 0.63), maternal education of 10 years and above (β = 0.15; 95% CI: 0.03, 0.27) and higher household wealth quintile were positively associated with WAZ at 3–5 months of life. Higher neonatal WAZ (β = 0.07, 95% CI: 0.03, 0.11), younger infant age (β=-0.11, 95% CI: -0.18, -0.04), and higher maternal BMI (β = 0.02, 95% CI: 0.01, 0.04) were positively associated with WLZ at 3–5 months of age.

## Discussion

We found no significant association between exposure to PLF and infant size at three to five months of age. However, infants who were exposed to PLF tended to have higher LAZ and lower WLZ and WAZ at three to five months of age. Female sex, larger size during the neonatal period, higher maternal education, and household wealth were associated with better size outcomes.

A possible reason for the null finding in our study might be because we only examine the attained size at 3 months of age, we did not capture growth interval. There may be more subtle differences in growth trajectories with PLF, such as changes in Z-score between birth and 3 months of age. There might be another intermediate risk factor like morbidity in the pathway between PLF and infant size. In addition, we assessed anthropometric measures around 3-month postpartum. We did not have data on infant size at an earlier time point, such as 1- month postpartum. It is also possible that infants in the study were exposed to pathogens through other sources in their environment, which might have attenuated the association between PLF and growth. One study in India also found an insignificant association between exposure to PLF, defined as foods other than breastmilk on the first day of life, and undernutrition among children less than 12 months old [40]. But they only conducted bivariate analysis. Another study collected data from several South Asian countries and found that provision of PLF, defined as foods other than breastmilk within first 3 days of life, increased the risk of wasting and severe wasting [41].

The main challenge of comparing the prevalence of PLF over time in a consistent manner is that there is no standard language for the questions asked to obtain the information on the exposure. Different definitions and terminologies have been used to describe the exposure. Most studies defined the exposure as any feed other than breastmilk to child during the first three days of life [16, 20, 21, 42]. Some defined the exposure as any feed other than breastmilk to children immediately after birth [24]. A number of studies did not specify the definitions [43–45]. The terminologies used to describe the exposure also varied across studies; the most commonly used term to describe the exposure is “prelacteal feeding”. Since most studies that defined the exposure as “any feed other than breastmilk during the first three days of life” used “prelacteal feeding” to describe the exposure, we decided to use PLF in our study to be consistent with other literature. There has been growing attention to provide better clarity in wording and definition of early exposure to non-breastmilk feed [46].

The Bangladesh Demographic and Health Surveys (DHS) consistently asked the same question— “In the first three days after delivery, was (NAME) given anything to drink other than breast milk”, and it found the prevalence of PLF decreased from 62% in 2007 [47] to 27% in 2014 [48] and increased slightly to 29% in 2018 [49]. From the JiVitA studies, using other feeds than breastmilk in the first three days of life as definition, we also observed a declining trend from 89% from data collected in 2001–2007 [20] to 24% from data collected in 2018–2019 here. Multiple Indicators Cluster Survey (MICS) conducted in 2006 asked “Did you give honey/sugar water/mustard oil/other to your child immediately after birth” and found a prevalence of 61% [50]. Later MICSs asked the same question as DHS and found the prevalence to be 23% in 2013 [51]. The most recent MICS conducted in 2019 found the prevalence to be 24% [13]. JiVitA data, like national survey data, are consistent with a decline in the PLF over the past couple decades.

A previous JiVitA study [31], carried out between 2012 and 2013, found that the prevalence of stunting, wasting, and underweight at 6 months of life were 25%, 6%, and 20%, respectively. All three prevalence estimates (20% stunted; 4% wasted; and 16% underweight) were lower at the time of our data collection between 2018 and 2019. This was consistent with the overall improvement in child nutrition status in Bangladesh in the past decades [13, 49]. In the most recent MICS conducted in 2019, it was estimated that the prevalence of stunting, wasting, and underweight for children under age 5 were 28%, 9.8%, and 22.6%, respectively [13]. We observed a slightly better nutritional status among our study population possibly because the anthropometric measurements were taken at a younger age and the participants resided in a research site where multiple projects aiming to improve maternal and newborn health have been implemented. However, compared to the WHO standard, infant size was still undesirable, where mean LAZ, WLZ, and WAZ were all below zero. When comparing to other LMICs, the estimated national prevalence of stunting among children under five in Bangladesh is still considered very high [52].

A major strength of our study was the prospective study design with a large cohort of pregnant women recruited from the community. Unlike many other studies that asked PLF questions after a couple of months, or even years after birth, our study collected the information within 14 days after birth. The responses to the PLF were comparable between birth visits conducted within 3 days (22%) vs. anytime between 4 days to 14 days after birth (23%). And more than half of the interviews (53%) were done within 3 days postpartum, which minimized the recall bias and was more likely to capture the true feeding behavior. A limitation of the current study was that in the analysis, we combined all the different types of prelacteal foods and treated them as the same exposure. This indicates the assumption that all the different types of food exert the same effects, which might not be true.

## Conclusion

Prelacteal feeding is still a common practice in rural Bangladesh. Although no association between prelacteal feeding and infant size was identified within the current study, we cannot ignore the potential harm posed by PLF, both directly from environmental contaminants in non-breastmilk foods and indirectly through delaying early initiation of breastfeeding and continued non-exclusive breastfeeding. In general, a clearer definition and guidelines about PLF is necessary to make comparisons geographically and chronologically. Future studies could assess the infant size at an earlier time point, such as 1-month postpartum, or use longitudinal data to assess more subtle differences in growth trajectories with PLF.

### Electronic supplementary material

Below is the link to the electronic supplementary material.

Additional file 1: Flow chart of study participants included and excluded from the current analysis

## Data Availability

Data described in the manuscript, code book, and analytic code will be made available upon request pending application and approval.

## Abbreviations

DHS: Demographic and Health Survey
LAZ: Length-for-age Z-score
LMICs: Low- and middle-income countries
LSI: Living Standard Index
MD: Mean difference
MICS: Multiple Indicator Cluster Survey
PLF: Prelacteal feeding
SD: Standard deviation
WAZ: Weight-for-age Z-score
WLZ: Weight-for-height Z-score
