# Supplementary material for: Prelacteal feeding is not associated with infant size at 3 months in rural Bangladesh: a prospective cohort study
Source: Int Breastfeed J. 2024 Feb 27;19:15. doi: 10.1186/s13006-024-00621-4 (PMC10900540; doi:10.1186/s13006-024-00621-4)
Supplement: Supplementary file 1 — Additional file 1: Flow chart of study participants included and excluded from the current analysis [file 13006_2024_621_MOESM1_ESM.docx]

**Supplement Figure 1. Flow chart of study participants included and excluded from the current analysis.**

**
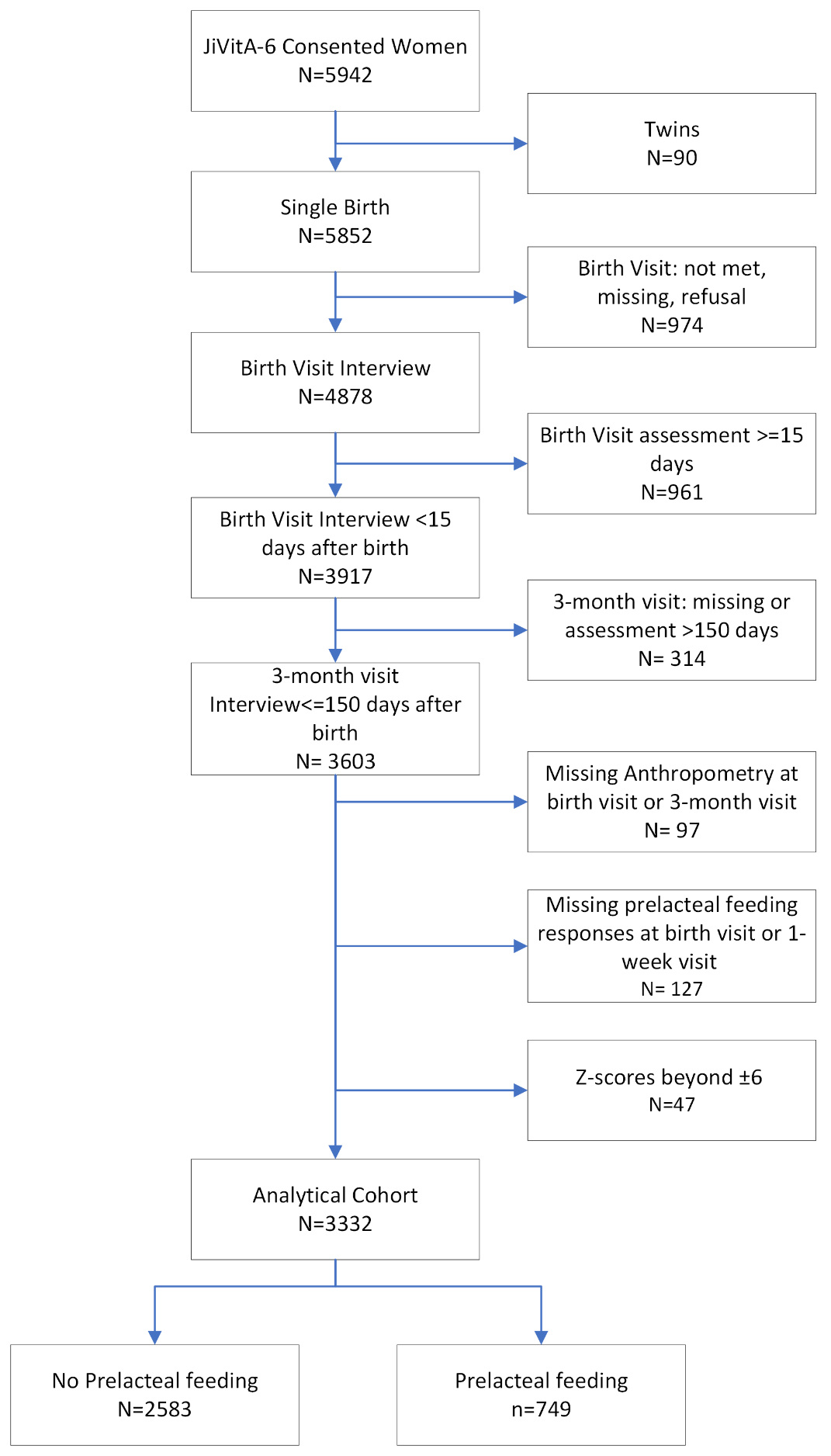
**
